# Genomic Analysis and Population Divergence Driven by Geographic Isolation in *Neotetracus sinensis*

**DOI:** 10.1002/ece3.73375

**Published:** 2026-04-05

**Authors:** Jianxuan Zhou, Nannan Chen, Weihao Dou, Zhonglai Luo, Libo Jiang, Meidong Jing, Fengtang Yang

**Affiliations:** ^1^ School of Life Sciences and Medicine Shandong University of Technology Zibo Shandong China; ^2^ College of Life Sciences Nantong University Nantong Jiangsu China

**Keywords:** chromosome‐level genome, genetic diversity, *Neotetracus sinensis*, population structure, PSMC/MSMC2 analyses

## Abstract

Mountain systems are natural laboratories for evolution, where rugged topography and ecological heterogeneity restrict gene flow. In the Hengduan Mountains, *Neotetracus sinensis* ( *N. sinensis* ), the sole species of its genus, occupies cool montane forests and has limited dispersal. Here we assemble a high‐quality reference genome and resequenced individuals from two isolated ranges in Yunnan, specifically Gaoligong Mountains (GLG) and Wuliang Mountains (WL), to test how geographic isolation shapes genomic variation. Genome‐wide SNPs reveal clear population structure, with PCA and phylogeny concordantly resolving two distinct clades. Relatedness analysis shows no close‐kin duplicates, and ROH are uniformly short, indicating outbred genomes. Demographic reconstructions show a shared trajectory marked by expansion ~1.0 Mya and a prolonged decline through the Middle‐Late Pleistocene. Despite a lower long‐term *N*ₑ, WL population displays contemporary nucleotide diversity (*π*), consistent with very recent gene flow or rapid post‐bottleneck recovery. To robustly assess genomic differentiation and mitigate biases from the small WL sample size, we employed a strict two‐tiered filtering approach. The intersection of the top 1% relative (*F*_st_) and absolute (*D*_xy_) divergence windows identified 5 core genomic islands, notably highlighting the DNA repair gene *MGMT* as a key candidate for environmental adaptation. Concurrently, functional enrichment of the broader top 1% *F*_st_ regions indicated an overrepresentation of pathways associated with synaptic and neuromodulatory signaling, cytoskeleton‐linked intracellular transport, and circadian regulation. These results provide a genomic framework for understanding how geographic isolation and montane environments shape divergence in *N. sinensis* .

## Graphical abstract

We generated the first chromosome‐level reference genome for *Neotetracus sinensis* and resequenced populations from the Gaoligong and Wuliang Mountains to investigate divergence across the Hengduan “sky‐island” landscape. Genomic data reveal strong population structure with negligible gene flow and parallel demographic histories shaped by Pleistocene climatic shifts. Divergence is concentrated in discrete genomic regions enriched for pathways related to neural signaling and circadian regulation, providing testable hypotheses for adaptation to heterogeneous montane environments.

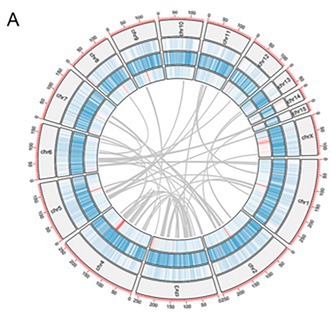

## 1 Introduction

Mountain ecosystems, with their complex topography, steep climatic gradients, and mosaic habitats, are among the most important drivers of biodiversity and diversification. The Hengduan Mountains of southwestern China exemplify this phenomenon, as they are characterized by deeply dissected valleys and serrated ridgelines and harbor exceptionally high levels of species richness and endemism (Gong et al. 2001). In this “sky‐island” landscape, steep environmental gradients and rugged terrain act as semi‐permeable barriers that restrict gene flow and promote population divergence, especially in organisms with limited dispersal capabilities (Zeng et al. 1994). Such features have produced a remarkable biogeographic mosaic, making the Hengduan Mountains an ideal natural laboratory for investigating how geographic complexity shapes evolutionary processes.

Among characteristic fauna of this region, the shrew gymnure, *Neotetracus sinensis* ( *N. sinensis* ), represents a relict lineage and the sole extant member of its genus. Distributed across southwestern China, northeastern Myanmar and northern Vietnam, *N. sinensis* typically inhabits subtropical evergreen and montane rainforests at elevations of 1500–2700 m. As a strictly terrestrial and nocturnal insectivore with low dispersal capacity, it is highly sensitive to habitat fragmentation and topographic isolation (Wilson and Mittermeier 2018). Although currently classified as Least Concern by the IUCN (Smith and Johnston 2016), increasing anthropogenic disturbances within its range underscore the need for a comprehensive understanding of its population structure and evolutionary history, with important implications for biodiversity conservation in the Hengduan region.

Previous work reported the complete mitochondrial genome of *N. sinensis* and clarified its placement within Erinaceidae (Lu et al. 2013). Phylogenetic analyses of 12 concatenated protein‐coding genes indicate that Galericinae diverged before Erinaceinae and that *N. sinensis* represents a distinct genus, splitting after *Echinosorex gymnura* . However, mitochondrial data alone provide limited insight into genome‐wide variation, population structure, or adaptive divergence. In particular, little is known about how geographic barriers and environmental heterogeneity across the species' fragmented range have influenced its evolutionary history. Against this backdrop, *N. sinensis* provides an ideal model for investigating how landscape barriers shape divergence in montane small mammals across multiple geographically isolated mountain systems.

Despite its ecological significance, the extent to which long‐term geographic isolation and environmental heterogeneity have shaped genetic divergence in *N. sinensis* remains largely unknown. To address this gap, we assembled a high‐quality reference genome and resequenced 10 individuals from two geographically isolated populations in Yunnan. The first, in the Wuliang Mountains (WL) of central Yunnan, is an uplifted plateau that has been deeply incised by the Lancang and Yuanjiang Rivers. The second is the Gaoligong Mountains (GLG), a north–south‐oriented range within the Hengduan system. The two ranges lie approximately 400 km apart and occupy contrasting geological and ecological settings, providing a natural experiment for investigating the evolutionary history and population divergence of this montane insectivore. Using a genome‐wide SNP dataset, we integrated population‐genomic, phylogenetic, and demographic analyses to characterize patterns of divergence between the ranges, while PSMC and MSMC2 placed these patterns in a historical context of effective population size change. We further identified highly differentiated genomic regions and assessed their functional enrichment to explore potential adaptive processes. Together, these approaches enable a comprehensive assessment of how complex montane landscapes shape genomic structure and evolutionary dynamics in this relict mammal.

## 2 Materials and Methods

### 2.1 Ethics Statement

All animal sampling and experimental procedures in this study were conducted in accordance with the Shandong University of Technology Laboratory Management Regulations (Document No. 163) and were supervised by the relevant institutional authorities.

### 2.2 Sampling and Sequencing

Species identity was confirmed by external morphological characters following Corbet and Hill (1992). Hind‐limb skeletal muscle was sampled in the field, preserved in absolute ethanol (100%), and stored at −20°C in the laboratory. We collected 10 individuals of *N. sinensis* from two geographically isolated mountain systems in Yunnan Province, China: the WL (*n* = 3) and the GLG (*n* = 7). One individual from WL was selected for the de novo genome assembly. High‐quality PacBio HiFi sequencing (~24× coverage) was performed, and chromosome‐scale scaffolding was achieved using Hi‐C data (Wenger et al. 2019). The remaining nine individuals (WL, *n* = 2; GLG, *n* = 7) were sequenced with Illumina sequencing, with an average depth of ~10× per individual. Raw PacBio HiFi read metrics were calculated directly from the unfiltered FASTQ files using SeqKit v2.9.0 (Shen et al. 2016).

### 2.3 Genome Size Estimation

Before assembly, we estimated genome size from the PacBio HiFi reads using a k‐mer approach. Canonical 21‐mers were counted with Jellyfish v2.3.1 (parameters: ‐C ‐m 21) and a k‐mer frequency distribution was generated using jellyfish histo (Marçais and Kingsford 2011). The frequency distribution was then modeled in R v4.4.1 with findGSE v1.9.4, which filters out low‐frequency error *k*‐mers (Sun et al. 2018).

### 2.4 Genome Assembly and Evaluation

PacBio HiFi reads were assembled de novo with hifiasm v0.24.0 to generate primary contigs (Cheng et al. 2024). Haplotypic duplications were removed using purge_dups v1.2.5 based on read‐depth histograms from minimap2 v2.28 (parameters: ‐x map‐hifi) (Guan et al. 2020; Li 2021). Hi‐C reads were aligned with Omni‐C pipeline following the recommended workflow, and contigs were scaffolded with YaHS v1.2.2, followed by manual curation in Juicebox v1.11.08 (Durand et al. 2016; Zhou et al. 2023). The final Hi‐C contact heatmap (Figure S2) was generated and visualized using HiCExplorer v3.7.6 (Ramírez et al. 2018). Remaining gaps were polished with TGS‐GapCloser v1.2.1 to improve assembly contiguity (Xu et al. 2020). HiFi reads were mapped back to the assembly with minimap2, and alignment statistics (mapping rate, depth, and coverage) were calculated using samtools v1.21 (Li et al. 2009). Consensus accuracy was estimated with Merqury v1.3 by comparing read‐derived *k*‐mers with those in the assembly, yielding QV, per‐base error rate, and completeness metrics (Rhie et al. 2020). Functional completeness was evaluated with BUSCO v5.4.6 against the mammalia_odb10 dataset (Simão et al. 2015).

### 2.5 Gene Prediction

Repetitive elements were identified in two steps. First, RepeatModeler2 v2.0.5 was used for de novo discovery and classification of repeat families to generate a species‐specific library (Flynn et al. 2020). Next, RepeatMasker v4.1.6 with RMBlast combined this library with the Dfam v3.8 database to annotate repeats genome‐wide, producing masked assemblies and class‐level summaries. Soft‐masked sequences (‐xsmall) were used for downstream analyses, and Kimura divergence was calculated from alignment files (Kimura 1980; Storer et al. 2021; Tarailo‐Graovac and Chen 2009). Gene prediction was performed with ANNEVO v2.1 in deep‐learning ab initio mode using default pretrained models (Ye et al. 2025). Gene completeness was evaluated with BUSCO v5.4.6 (mammalia_odb10, protein mode). The quality of gene prediction was further assessed using OMArk v0.3.1 with OMAmer against the Laurasiatheria reference, summarizing ortholog recovery and classifying predicted proteins (Nevers et al. 2025). After evaluating gene models, we proceeded to annotate non‐coding RNA families. tRNAs were identified using tRNAscan‐SE v2.0.12 (eukaryotic mode), rRNAs with barrnap v0.9, and other small ncRNAs (snRNAs, snoRNAs, miRNAs) with Infernal v1.1.5 against the Rfam database (release 15.0) (Chan et al. 2021; Griffiths‐Jones et al. 2003; Nawrocki and Eddy 2013; Seemann, n.d.).

### 2.6 Functional Annotation

Protein sequences were first exported from the structural annotation (GFF3 + genome FASTA) using gffread v0.12.7 to obtain the CDS and translated proteins (Pertea and Pertea 2020). We assigned orthology‐based terms with eggNOG‐mapper v2.1.13 (yielding eggNOG, GO and KEGG results) (Cantalapiedra et al. 2021; Huerta‐Cepas et al. 2019; Kanehisa and Goto 2000; The Gene Ontology Consortium 2021). Protein domains and families were identified with InterProScan v5.60‐92.0 (Jones et al. 2014), including the PANTHER and Pfam member databases (Mi et al. 2021; Mistry et al. 2021). Curated similarity was obtained by searching proteins against UniProtKB (The UniProt Consortium 2023), and NCBI nr was used as additional evidence (Sayers et al. 2022).

### 2.7 Genome Landscape and Synteny Visualization

GC content, gene density, and repeat density were summarized on the soft‐masked assembly using 1‐Mb non‐overlapping windows. GC content was calculated with bedtools v2.31.1 (Quinlan and Hall 2010), gene counts were derived from the GFF annotation, and repeat coverage was obtained from RepeatMasker outputs. Syntenic blocks were identified by first conducting an all‐versus‐all protein similarity search with DIAMOND v2.1.8.162 (BLASTP mode; e‐value ≤ 1 × 10^−5^) using the longest isoform per gene (Buchfink et al. 2021). The resulting hits were analyzed with MCScanX (Wang et al. 2012), requiring ≥ 5 collinear gene pairs and a maximum gap of 25 genes. Circular plots were generated with TBtools v2.147 (Chen et al. 2020).

### 2.8 Population Genomic Analyses

#### 2.8.1 Read Processing and Alignment

Raw resequencing reads were filtered and adapter‐trimmed with fastp v0.24.0 (Chen et al. 2018). Clean reads were aligned to the WL reference genome using BWA‐MEM v0.7.18 (Li and Durbin 2009), and alignments were sorted and deduplicated with samtools v1.21. Mapping statistics were obtained with samtools flagstat, while mean depth and coverage breadth were calculated from per‐base coverage profiles generated by samtools depth.

#### 2.8.2 Autosomal SNP Discovery and Filtering

To focus on autosomal variation, the X chromosome was excluded from the reference using seqkit. SNPs were jointly genotyped with bcftools v1.2.1 (mpileup ‐Q 20 ‐q 30 | call ‐mv) (Danecek et al. 2021). After left‐normalization and multiallelic splitting, biallelic autosomal SNPs were retained following hard filtering based on site quality, depth, minor allele frequency (MAF), and missingness.

#### 2.8.3 Population Structure Analyses

Linkage disequilibrium (LD) pruning was performed in PLINK v1.9.0 (‐‐indep‐pairwise 50 10 0.2), excluding variants with MAF < 0.05 or missingness > 0.10 (Purcell et al. 2007). Principal component analysis (PCA) was conducted in PLINK (‐‐pca), and results were visualized in R v4.4.1 with ggplot2. Pairwise relatedness was estimated from LD‐pruned SNPs using PLINK ‐‐genome. Pairwise relatedness (PI_HAT) from PLINK was visualized as a heatmap with pheatmap v1.0.12 (R) (Kolde 2019). Similarity was converted to dissimilarity as 1‐PI_HAT and rows/columns were hierarchically clustered using Euclidean distance and average‐linkage.

#### 2.8.4 Phylogenetic Inference

Filtered SNPs were converted to PHYLIP format with vcf2phylip v2.0 and used to infer a maximum‐likelihood phylogeny in IQ‐TREE v2.4.0 under the GTR + G model, with 1000 ultrafast bootstrap and SH‐aLRT replicates for node support (Nguyen et al. 2015; Ortiz 2019). Ascertainment bias correction was not applied due to the absence of invariant sites, and branch lengths were not interpreted. Trees were visualized as cladograms using ggtree v3.2.1 in R (Yu et al. 2017).

### 2.9 Linkage Disequilibrium (LD) Decay

LD decay was estimated from filtered, unpruned autosomal SNPs using PLINK v1.9 (‐‐*r* ^2^ ‐‐ld‐window 99999 ‐‐ld‐window‐kb 1000 ‐‐ld‐window‐*r* ^2^ 0), restricting sites to MAF ≥ 0.10. Pairwise *r* ^2^ values within 1 Mb were averaged in 10‐kb distance bins, and mean *r* ^2^ per bin was visualized in R with a LOESS fit.

### 2.10 Demographic History

Historical effective population size (*N*ₑ) was reconstructed with PSMC v0.6.5 and MSMC2 v2.1.4. Diploid consensus sequences were generated for each individual and converted to PSMC input using fq2psmcfa ‐q20 (Li and Durbin 2011; Schiffels and Wang 2020). PSMC was run with the default time pattern (−p 4 + 25 * 2 + 4 + 6) and 100 bootstrap replicates. MSMC2 used the same filtered SNP set as population analyses, with callable masks and multihetsep files generated using msmc‐tools. Both methods were scaled using a per‐generation mutation rate of *μ* = 6.0 × 10^−9^ mutations per site and a generation time of 3 years, following IUCN generation‐length guidance (Smith and Johnston 2016). This mutation rate lies within the empirically supported range for small vertebrates with similar generation times. It is consistent with pedigree‐based estimates for laboratory mice (Milholland et al. 2017) and close to the germline rate reported for zebra finch (5.0 × 10^−9^ per site per generation), which is on par with mammals that have a generation time of ~2–3 years (Prentout et al. 2025). Because no species‐specific mutation rate is available for *Neotetracus*, we treat *μ* as an approximate scaling factor that primarily affects the absolute time axis rather than the relative shape of the demographic trajectories. Individual trajectories were interpolated onto a common time grid and averaged per population for visualization in R v4.4.1.

### 2.11 Genomic Differentiation and Functional Enrichment

Genome‐wide genetic differentiation was estimated as Weir‐Cockerham *F*_st_ using VCFtools v0.1.16 with 50‐kb windows and 10‐kb steps (Danecek et al. 2011). The empirical top 1% of windows was defined as high‐divergence outliers, and adjacent outlier windows (< 10 kb apart) were merged into contiguous regions with bedtools. Genes overlapping these regions were extracted from the GFF3 annotation. Functional enrichment was performed by assigning KEGG orthologs and GO terms with eggNOG‐mapper v2.1.13 and testing pathway or term over‐representation with one‐sided Fisher's exact tests implemented in clusterProfiler (Yu et al. 2012).

Furthermore, to rigorously validate these differentiation signals and mitigate potential biases arising from the small sample size in the WL population, we calculated absolute divergence (*D*_xy_) across the same 50‐kb windows using pixy v2.0.0 (Korunes and Samuk 2021). The intersection of the empirical top 1% windows for both *F*_st_ and *D*_xy_ was subsequently extracted to represent the most robust, conservatively defined “core genomic islands” of differentiation. Genes overlapping these core islands were manually inspected to identify key candidates under strong divergent selection.

## 3 Results

### 3.1 Chromosome‐Scale Genome Assembly, Annotation and Functional Characterization of *N. sinensis*

**FIGURE 1:**
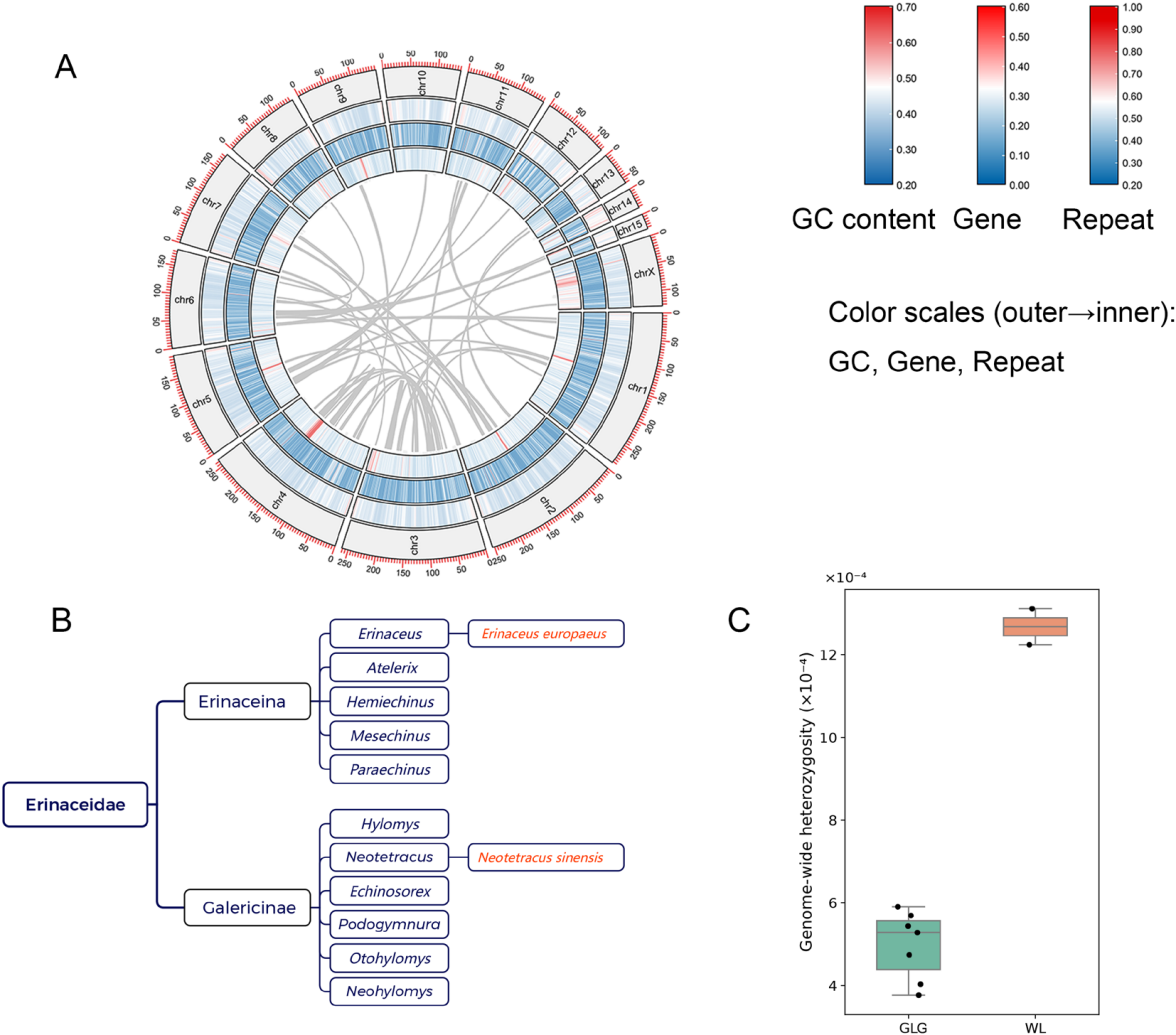
Genomic variation and population genetic diversity of *Neotetracus sinensis* along the 15 autosomes (chr1–chr15). (A) Circos view of the chromosome‐level assembly. From outside to inside: Pseudochromosome ideograms with Mb ticks, GC content, gene density, and repeat density (all summarized in 1‐Mb non‐overlapping windows). Gray ribbons mark intragenomic syntenic blocks inferred with MCScanX. (B) Taxonomic context within Erinaceidae. The position of *N. sinensis* (highlighted) within Galericinae is shown alongside genera in Erinaceinae. (C) Genome‐wide heterozygosity per individual for the GLG (*n* = 7) and WL (*n* = 2) populations. Boxes show IQR and median; points are individuals.

We generated a chromosome‐scale reference genome for *N. sinensis* using a representative individual from the WL population. K‐mer analysis estimated the genome size at 2.37 Gb with a repeat content of 25.5% (Figure S1). A total of 20.7 Gb of PacBio HiFi reads were used for de novo assembly (Table S1). The final assembly measured 2.43 Gb with a contig *N*50 of 14.89 Mb, indicating high sequence continuity. After Hi‐C scaffolding and gap filling, we reconstructed 16 pseudochromosomes (15 autosomes and chrX) consistent with the expected karyotype (2*n* = 32; Figure 1A). The Hi‐C contact map exhibited a clear diagonal pattern with minimal off‐diagonal noise, confirming the accurate ordering and orientation of these chromosome‐scale scaffolds (Figure S2). The scaffold N50 reached 179.99 Mb, highlighting the high contiguity of the assembly. Quality metrics supported high base‐level accuracy and completeness. Back‐mapping of HiFi reads yielded a 100% alignment rate, 24× mean depth, and 99.99% coverage breadth. K‐mer analysis with Merqury reported a consensus quality value (QV) of 61.3 and 98.2% k‐mer completeness. Gene‐space completeness was similarly high: BUSCO (mammalia_odb10) identified 95.4% complete orthologs (93.8% single‐copy, 1.6% duplicated), with 0.9% fragmented and 3.7% missing among 9226 BUSCO groups (Table S2).

Repeat sequences comprise a substantial portion of the *N. sinensis* genome, dominated by retroelements (~1.12 Gb; 43.8%). Among these, SINEs (~29.1%) are more abundant than LINEs (~13.4%), while LTR elements contribute minimally (~1.3%). DNA transposons are rare (~0.5%), and unclassified repeats account for ~5% (Table S3). Based on the soft‐masked assembly, we predicted 25,548 protein‐coding genes. BUSCO identified 92.7% complete orthologs (91.5% single‐copy, 1.2% duplicated), with 3.3% fragmented and 4.0% missing (Table S4A). Consistently, OMArk recovered 86.8% single‐copy and 10.1% duplicated HOGs (Table S4B). At the proteome level, OMArk “consistency” metrics showed that 90.9% of proteins were consistent (2.9% inconsistent; 0 contaminants), and best‐hit composition was dominated by *Erinaceus europaeus* (93.84%), the closest well‐annotated erinaceid reference (Table S4C). This pattern accords with the phylogenetic placement of *N. sinensis* within Erinaceidae (Figure 1B) and indicates minimal contamination. Together, BUSCO and OMArk metrics support a high‐quality annotation suitable for downstream comparative and population genomic analyses.

We annotated 5000 non‐coding RNA loci, including 3191 snRNAs, 1039 tRNAs, 345 rRNAs, 309 miRNAs, and 109 lncRNAs (Table S5). To illustrate chromosome‐scale features, a Circos plot (Figure 1A) summarizes pseudochromosomes, GC content, gene density, repeat density, and intragenomic synteny. We observe a complementary distribution between gene density and repeat density, indicative of heterogeneous genome architecture. Nearly all predicted proteins received functional assignments. eggNOG mapped 24,458 (95.73%) proteins, with GO terms for 20,095 (78.66%) and KEGG pathways for 18,578 (72.72%). Domain annotation covered 23,102 (90.43%) proteins by InterProScan, including PANTHER 22,497 (88.06%) and Pfam 21,183 (82.91%). Curated matches were found for 23,379 (91.51%) proteins in UniProt, with 7648 (29.94%) supported by NCBI nr (Table S6).

Overall, these results establish a chromosome‐scale, high‐accuracy reference genome for *N. sinensis* . The repeat‐rich architecture (~44% retroelements) and well‐supported annotation (25,548 protein‐coding genes and a diverse ncRNA landscape), and ~99% of proteins received at least one functional assignment, providing a robust foundation for downstream population genomics, demographic inference, and studies of adaptive divergence.

### 3.2 Genomic Variation and Genetic Diversity

Whole‐genome resequencing was conducted for nine individuals from two Yunnan populations: GLG (*n* = 7) and WL (*n* = 2) (IDs: GLG = G; WL = wls). Each library produced 21.9–34.8 Gb of clean reads (median ~30.5 Gb) with mean depths of 7.0–11.2×. Mapping rates (97.36%–98.71%) and genome coverage (97.13%–99.62% at ≥ 1×) confirmed high‐quality data suitable for SNP discovery (Table S7). Levels of genome‐wide variation differed markedly between populations. WL individuals carried 2.83–3.03 million heterozygous SNPs per genome, approximately two to three times higher than in GLG individuals (0.87–1.36 million). Consistently, autosomal per‐site heterozygosity was elevated in WL (1.22–1.31 × 10^−3^) relative to GLG (0.38–0.59 × 10^−3^) (Figure 1C; Table S8). To further evaluate population‐level genetic variation, we calculated nucleotide diversity (*π*), which yielded consistent results: the *π* value was substantially higher in the WL population (6.54 × 10^−4^) compared to the GLG population (1.79 × 10^−4^). Although the WL sample size is small (*n* = 2), the pronounced effect size strongly indicates substantially greater standing genetic diversity in this population.

These results demonstrate pronounced population‐level differences in genetic variation between the two mountain systems. The WL population retains significantly greater genetic diversity than the GLG population, consistent with long‐term geographic isolation and distinct demographic trajectories shaping their evolutionary histories.

### 3.3 Population Structure and Genetic Differentiation

**FIGURE 2:**
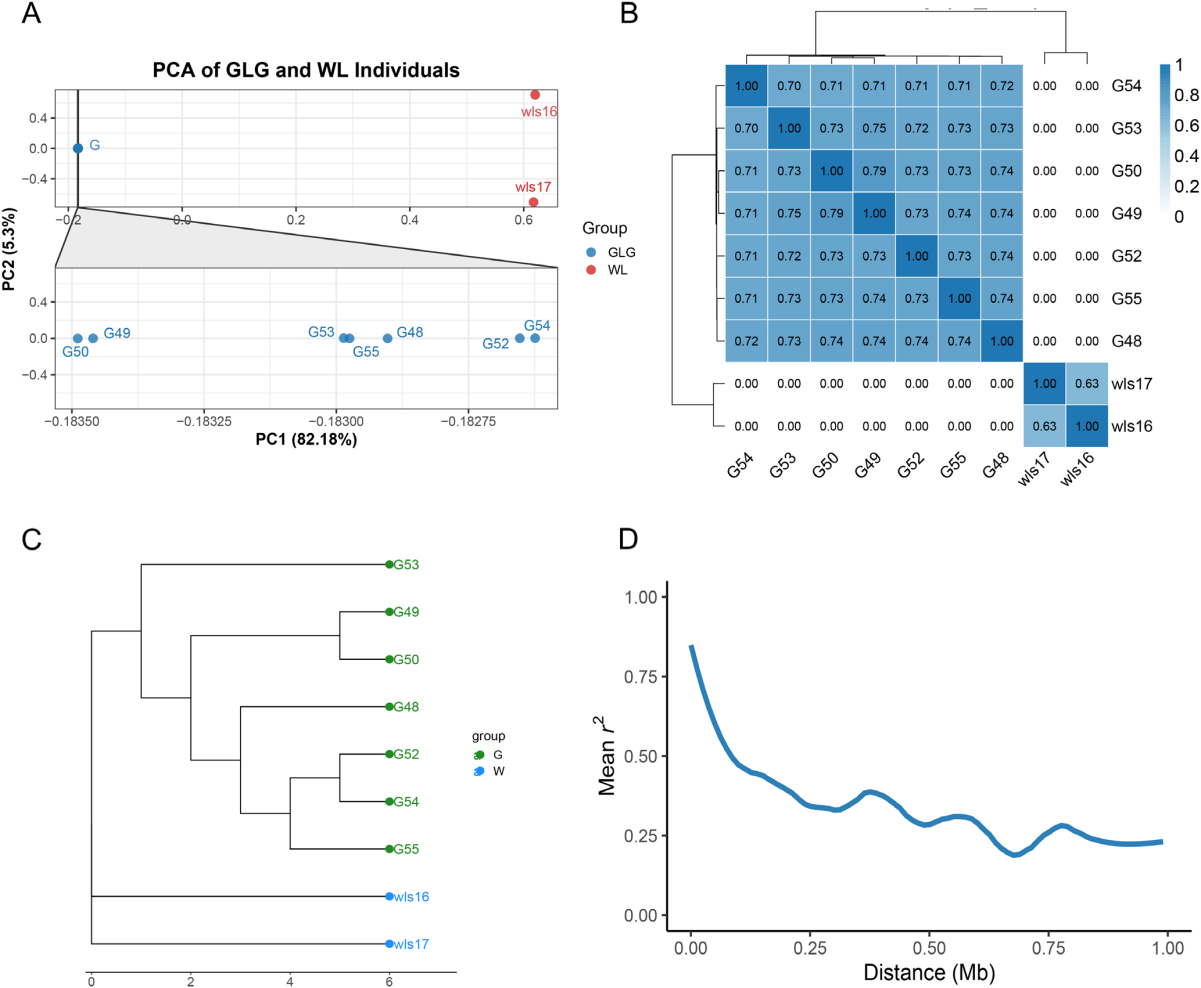
Population structure of the GLG and WL populations revealed by SNP analyses. (A) Principal component analysis (PCA) based on 53,972 SNPs after linkage disequilibrium pruning. The inset in panel A provides a zoomed‐in view of the GLG individuals to better illustrate their internal genetic distribution. (B) Identity‐by‐descent (IBD) heatmap based on PI_HAT values. (C) Phylogenetic tree constructed using SNP data. (D) LD decay by population. Mean pairwise *r* ^2^ is plotted against physical distance (Mb).

Principal component analysis (PCA) based on 53,972 LD‐pruned SNPs revealed clear population structure among the nine re‐sequenced individuals (Figure 2A). PC1 explained 82.18% of the total variance and separated the GLG and WL populations, indicating strong geographic differentiation. PC2 (15.36%) further distinguished the two WL individuals, suggesting potential within‐population heterogeneity. Identity‐by‐descent (IBD) analysis supported these findings (Figure 2B). Within the GLG population, high pairwise PI_HAT values (0.70–0.79) indicated close genetic relationships and likely recent shared ancestry. The two WL individuals showed a moderately lower IBD value (0.63), consistent with more distant kinship. No IBD sharing was detected between populations, demonstrating limited gene flow and a strong genetic barrier. Phylogenetic reconstruction further corroborated these results (Figure 2C). All GLG individuals clustered into a well‐supported clade, while the two WL samples formed a distinct lineage. Their separation within this clade, as well as along PC2, indicates genetic heterogeneity within WL despite their shared geographic origin. PCA, IBD, and phylogenetic analyses consistently demonstrate pronounced genetic divergence between the GLG and WL populations, shaped by long‐term isolation and restricted gene flow.

### 3.4 Linkage Disequilibrium (LD) Decay

Mean LD decayed rapidly in the GLG population (Figure 2D). Pairwise *r* ^2^ started around 0.80 at short distances and declined below 0.50 by ~0.15–0.20 Mb, indicating a relatively short half‐decay distance. LD continued to decrease to ~0.30 by ~0.5 Mb and reached a plateau of ~0.20–0.25 beyond 0.8–1.0 Mb. In contrast, the WL population showed *r* ^2^ ≈1.0 across all distances due to the extremely small sample size (*n* = 2), rendering LD estimates uninformative. The rapid decay observed in GLG is consistent with weak long‐range LD, shorter haplotype blocks, and a comparatively larger long‐term effective population size.

PCA, IBD, and phylogenetic analyses all demonstrate strong genetic differentiation between the GLG and WL populations with minimal contemporary gene flow. Within GLG, modest structure reflects recent relatedness among the sampled individuals, whereas inferences about within‐population structure in WL are limited by its very small sample size. LD patterns further support this view: GLG exhibits clear LD decay, while WL LD estimates cannot be meaningfully interpreted. These results support treating GLG and WL as genetically differentiated units for subsequent analyses.

### 3.5 Demographic History of *N. sinensis*

**FIGURE 3:**
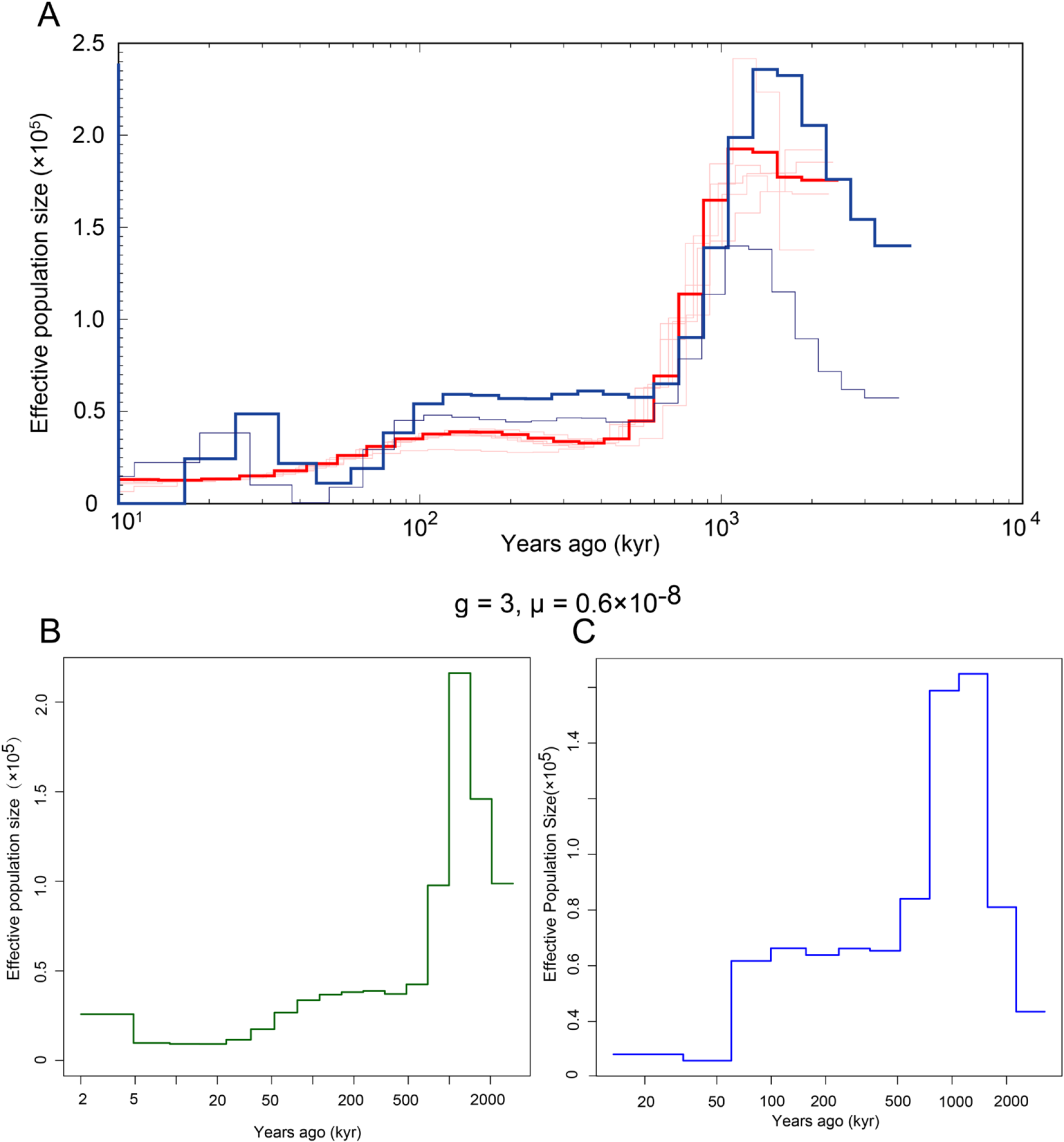
Inference of historical effective population size (*N*_e_) in *Neotetracus sinensis* populations. (A) Effective population size trajectories inferred using PSMC for two populations: GLG group (red) and WL group (blue). Each line represents the *N*_e_ trajectory from a single diploid individual. Thick lines denote average trends across individuals in each group. The X‐axis shows years before present (assuming generation time = 3 years, mutation rate *μ* = 6 × 10^−9^ per site per generation); the Y‐axis indicates effective population size (in 10^4^ individuals). (B) Effective population size history of the GLG group, inferred using MSMC2. (C) Effective population size history of the WL group, inferred using MSMC2.

We reconstructed historical changes in effective population size (*N*_e_) using PSMC based on eight high‐coverage genomes (Figure 3A). Both populations exhibited broadly similar demographic trajectories. An extended period of population growth began more than 1 million years ago, with *N*_e_ reaching approximately 2.2 × 10^5^ in GLG and 1.5 × 10^5^ in WL around ~1.0 Mya. This pattern is broadly consistent with the environmental heterogeneity generated by major orogenic processes in the region, although precise correspondence should be interpreted with caution given the temporal uncertainty inherent in PSMC estimates. A subsequent decline in *N*_e_ between ~300 and 100 kyr overlapped with climatic fluctuations during the Middle Pleistocene, which may have reduced suitable habitats for *N. sinensis* . Over the past 100 kyr, inferred *N*_e_ remained relatively low (~0.5–0.8 × 10^5^), though recent intervals cannot be resolved reliably due to methodological limits of PSMC at shallow timescales. Complementary MSMC2 analyses (Figure 3B,C) were employed to better resolve these recent dynamics. The results recovered similar qualitative patterns, showing population increase prior to ~300 kyr followed by more recent declines. Across methods, the GLG population tended to exhibit higher inferred *N*_e_, consistent with historically larger or more persistent population sizes in this region, although these differences should be interpreted cautiously. All demographic models were scaled using a per‐site mutation rate of *μ* = 6 × 10^−9^ per generation and a generation time of 3 years.

### 3.6 Genomic Differentiation and Functional Enrichment

**FIGURE 4:**
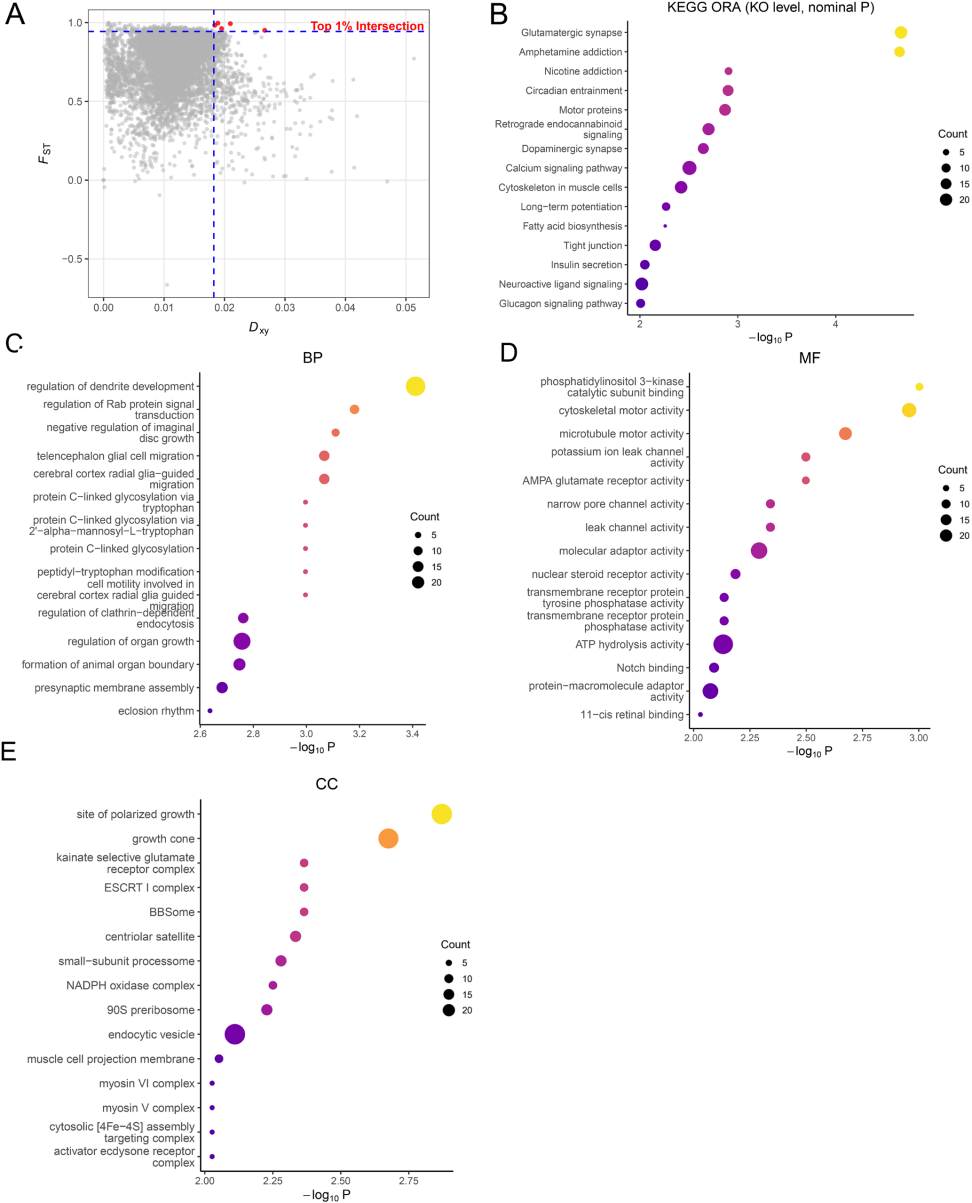
Genome‐wide differentiation and functional enrichment of outlier genes. (A) 2D scatter plot showing genome‐wide relative (*F*_st_) and absolute (*D*_xy_) differentiation calculated in 50‐kb windows. The horizontal and vertical dashed lines mark the empirical 99th percentiles (top 1%) for *F*_st_ and *D*_xy_, respectively. The red points in the top‐right quadrant highlight the 5 core genomic islands situated at the intersection of both top 1% thresholds. (B) KEGG over‐representation at the KO level for genes overlapping the broader merged top 1% *F*_st_ outlier regions (universe = all KO‐annotated genes). One‐sided hypergeometric tests were used and *p* values are reported (no multiple‐testing correction). The bubble plot is ranked by *p*; the x‐axis shows –log_10_(*P*), bubble size denotes the number of hits (Count), and color encodes –log_10_(*p*). The panel displays the top pathways meeting *p* < 0.10. (C–E) GO over‐representation for BP (C), MF (D), and CC (E) using the same settings (one‐sided hypergeometric; *p* values only; terms with < 5 genes excluded; universe = all GO‐annotated genes). Axes and symbols as in (B); each panel shows terms with *p* < 0.10 (or the top N if fewer).

We next quantified genome‐wide differentiation and examined functional clustering of candidate genes. Genome‐wide *F*_st_ analysis revealed a right‐skewed distribution with discrete peaks of elevated differentiation. To strictly validate these signals and mitigate potential biases from the small WL sample size, we intersected the top 1% *F*_st_ windows with the top 1% absolute divergence (*D*_xy_) windows (Figure 4A, Figure S3). This extremely stringent dual‐threshold approach identified 5 core genomic islands representing the most robust loci of divergence. Manual inspection of these core windows revealed a single, highly compelling candidate gene: MGMT (chr3‐g1237), which encodes a methylated‐DNA‐protein‐cysteine S‐methyltransferase essential for direct DNA repair and genomic stability under environmental stress.

Using the empirical 99th percentile as a threshold and merging adjacent windows ≤ 10 kb apart, we identified 845 high‐divergence regions (median size: 60.0 kb; maximum: 270.0 kb). Intersecting these regions with gene annotations yielded 759 unique candidate genes for downstream analyses.

KEGG enrichment analysis revealed two broad functional clusters (Figure 4B): (i) synaptic signaling and neuromodulatory pathways, including components of Ca^2+^‐ and cAMP‐linked PKA/ERK/CREB cascades, and (ii) cytoskeleton‐ and motor‐associated intracellular transport processes involving microtubule/actin systems and dynein/kinesin/myosin families. GO enrichment results were highly consistent with these themes (Figure 4C–E), highlighting terms related to synaptic structure and transmission, plasticity‐associated processes, and membrane trafficking and cytoskeletal–motor systems. Although the high‐divergence regions represent a small fraction of the genome and enrichment signals are modest, both KEGG and GO analyses indicate that genes involved in synaptic function, neuromodulatory pathways, and intracellular transport are disproportionately represented. These functional patterns provide a basis for generating hypotheses about potential targets of selection in *N. sinensis* , particularly those related to neural, behavioral, or locomotor traits in heterogeneous montane environments.

## 4 Discussion

Genome‐wide SNP analyses provide consistent evidence for deep population structure between the GLG and WL populations of *N. sinensis* . Principal component analysis clearly separated all individuals by range, identity‐by‐descent estimates revealed negligible relatedness across populations, and a maximum‐likelihood tree recovered two well‐supported clades corresponding to GLG and WL. Runs of homozygosity were rare and short in both groups, indicating low levels of recent inbreeding and confirming that our samples represent largely outbred genomes (Ceballos et al. 2018). These findings point to restricted gene flow and long‐term isolation between the two ranges, a pattern readily explained by geography. The two populations are separated by ~400 km of deep valley corridors and differ markedly in elevation, climate, and forest structure. For a small, forest‐dependent insectivore with limited dispersal, this classic “sky‐island” topography likely imposes a strong barrier to gene flow and promotes lineage divergence.

This pattern is consistent with observations from other Hengduan small mammals, including long‐tailed moles ( *Scaptonyx fusicaudus* ), plateau pikas ( *Ochotona curzoniae* ), and Yunnan red‐backed voles ( *Eothenomys miletus* ) (Ci et al. 2009; He et al. 2019; Ren et al. 2023). Across these taxa, deep genetic structure among adjacent mountain massifs, divergence times clustering around the Mid‐Pleistocene Transition, and restricted contemporary connectivity are repeatedly observed, with major rivers (Nu, Lancang, Jinsha), elevational gradients, and fragmented montane forests acting as semi‐permeable barriers. Our results thus align with a broader biogeographic model in which geographic complexity and ecological heterogeneity drive genetic subdivision in Hengduan mammals.

Demographic reconstructions further support this interpretation. Both PSMC and MSMC2 analyses reveal a shared demographic history marked by population expansion beginning ~1.0 Mya followed by a sustained decline through the Middle‐Late Pleistocene. The timing coincides with the Mid‐Pleistocene Transition (~1.2–0.7 Mya), suggesting that climatic regime shifts were likely key drivers of demographic dynamics (Li and Durbin 2011; Terhorst et al. 2017). The earlier uplift of the Hengduan Mountains during the late Miocene–Pliocene (10–3 Mya) likely set the heterogeneous topographic template but did not directly trigger population expansion. Rapid LD decay in the GLG population suggests a historically larger effective size, whereas higher present‐day genetic diversity in WL likely reflects more recent demographic processes such as post‐bottleneck recovery or localized gene flow, processes that are not captured by deeper coalescent histories (Browning and Browning 2015; Santiago et al. 2020).

Signals of genomic differentiation are highly localized. Crucially, our strict two‐tiered filtering approach—taking the intersection of the top 1% for both relative (*F*_st_) and absolute (*D*_xy_) divergence identified 5 core genomic islands representing the most robust signatures of evolutionary divergence. The discovery of the *MGMT* gene within these core islands is particularly noteworthy. As a key player in direct DNA repair, *MGMT* protects the genome against alkylating agents and environmental stress. Given the complex topography and environmental stressors associated with these high‐elevation Hengduan ranges, the strong divergence in *MGMT* points to localized adaptive shifts in mechanisms ensuring genomic integrity. Beyond these core islands, to capture the broader landscape of adaptation based on the wider top 1% *F*_st_ regions, KEGG over‐representation (KO level; one‐sided hypergeometric tests) highlighted two recurring functional themes when ranked by *p* value (display threshold *p* < 0.10): synaptic/neuromodulatory signaling and cytoskeleton‐linked intracellular transport/circadian regulation. These converge on Ca^2+^/cAMP–PKA/ERK–CREB cascades and microtubule/actin‐based motor systems, pointing to neural plasticity, behavioral responsiveness, vesicle/organellar trafficking, and daily timing as plausible axes of divergence in rugged montane habitats. GO over‐representation (BP/MF/CC), analyzed and reported by *p* value with the same display threshold, recovered categories consistent with these themes and serves as qualitative support.

Our study is not without limitations. The most significant is the small sample size from WL (*n* = 2), reflecting the inherent difficulty of field sampling for a nocturnal, forest‐dwelling insectivore in steep, high‐elevation habitats. Limited sampling reduces statistical power, inflates variance in *F*_st_, and precludes reliable LD‐ or haplotype‐based analyses. Nevertheless, our analytical framework effectively mitigates these concerns. Most importantly, the inclusion of absolute divergence (*D*_xy_) successfully filtered out regions where elevated *F*_st_ might be a mathematical artifact of low intra‐population diversity, allowing us to pinpoint genuine, deep evolutionary divergence (e.g., the *MGMT* locus). Furthermore, the main population structure signals are strong and consistent across independent methods, demographic trajectories align with geological and climatic history, and enrichment results are biologically coherent. Together, these considerations indicate that our findings remain robust and informative despite sampling constraints. Future efforts should aim to expand sampling (≥ 8–10 individuals per population at ≥ 15× coverage) to stabilize estimates of genetic diversity, improve power for haplotype‐based scans, and detect rare structural variants.

Looking ahead, three directions are especially promising. First, broader geographic sampling and deeper sequencing will enable landscape‐genomic analyses that explicitly link genetic variation to environmental gradients. Second, complementary demographic modeling using site frequency spectrum and multi‐individual coalescent frameworks will allow formal testing of alternative historical scenarios such as bottlenecks versus secondary contact (Excoffier et al. 2021; Gutenkunst et al. 2009). Third, integrating additional selection statistics with environmental association approaches and functional validation—such as transcriptomic profiling, mitochondrial performance assays, and recombination mapping—will help disentangle adaptive signals from background processes and directly connect genotype to phenotype (Forester et al. 2018).

In summary, this study assembled a chromosome‐scale reference genome for *N. sinensis* , documents deep population structure across major Hengduan barriers, and identifies genomic regions likely involved in montane adaptation. Although limited sample sizes constrain certain analyses, the demographic reconstructions and differentiation landscape are internally consistent and biologically meaningful. Our work provides a valuable foundation for future comparative and functional studies and contributes to a broader understanding of how complex mountain landscapes shape evolutionary trajectories in East Asian small mammals.

## Author Contributions

**Jianxuan Zhou:** formal analysis (lead), writing – original draft (lead). **Nannan Chen:** formal analysis (supporting), writing – original draft (supporting). **Weihao Dou:** writing – review and editing (equal). **Zhonglai Luo:** writing – review and editing (equal). **Libo Jiang:** methodology (equal), writing – review and editing (equal). **Meidong Jing:** investigation (lead). **Fengtang Yang:** methodology (equal), writing – review and editing (equal).

## Funding

This work was supported by the National Natural Science Foundation of China (Grant Nos. 32370689, 32070601, and 32471898).

## Conflicts of Interest

The authors declare no conflicts of interest.

## Supporting information


**Figures S1–S3:** ece373375‐sup‐0001‐Figures.docx.

**Tables S1–S8:** ece373375‐sup‐0002‐Tables.docx.

## Data Availability

The whole genome sequence data reported in this paper have been deposited in the Genome Warehouse in National Genomics Data Center (NGDC), Beijing Institute of Genomics, China National Center for Bioinformation, under accession number PRJCA051604 that is publicly accessible at https://ngdc.cncb.ac.cn/gwh.
