# Supplementary material for: Genomic Analysis and Population Divergence Driven by Geographic Isolation in Neotetracus sinensis
Source: Ecol Evol. 2026 Apr 5;16(4):e73375. doi: 10.1002/ece3.73375 (PMC13052205; doi:10.1002/ece3.73375)
Supplement: Supplementary file 1 — Figures S1–S3: ece373375‐sup‐0001‐Figures.docx. [file ECE3-16-e73375-s001.docx]

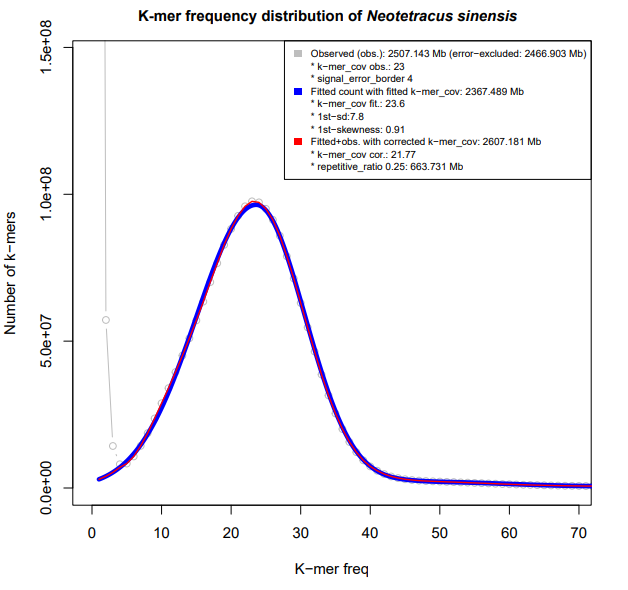


**Fig. S1. K-mer frequency distribution and genome size estimation of *Neotetracus sinensis*.**
A 21-mer histogram was generated using Jellyfish, and genome characteristics were inferred using findGSE. The main peak at ~23× represents the homozygous k-mer coverage. The estimated haploid genome size is approximately 2.37 Gb, with a repeat content of 25.5%.

**
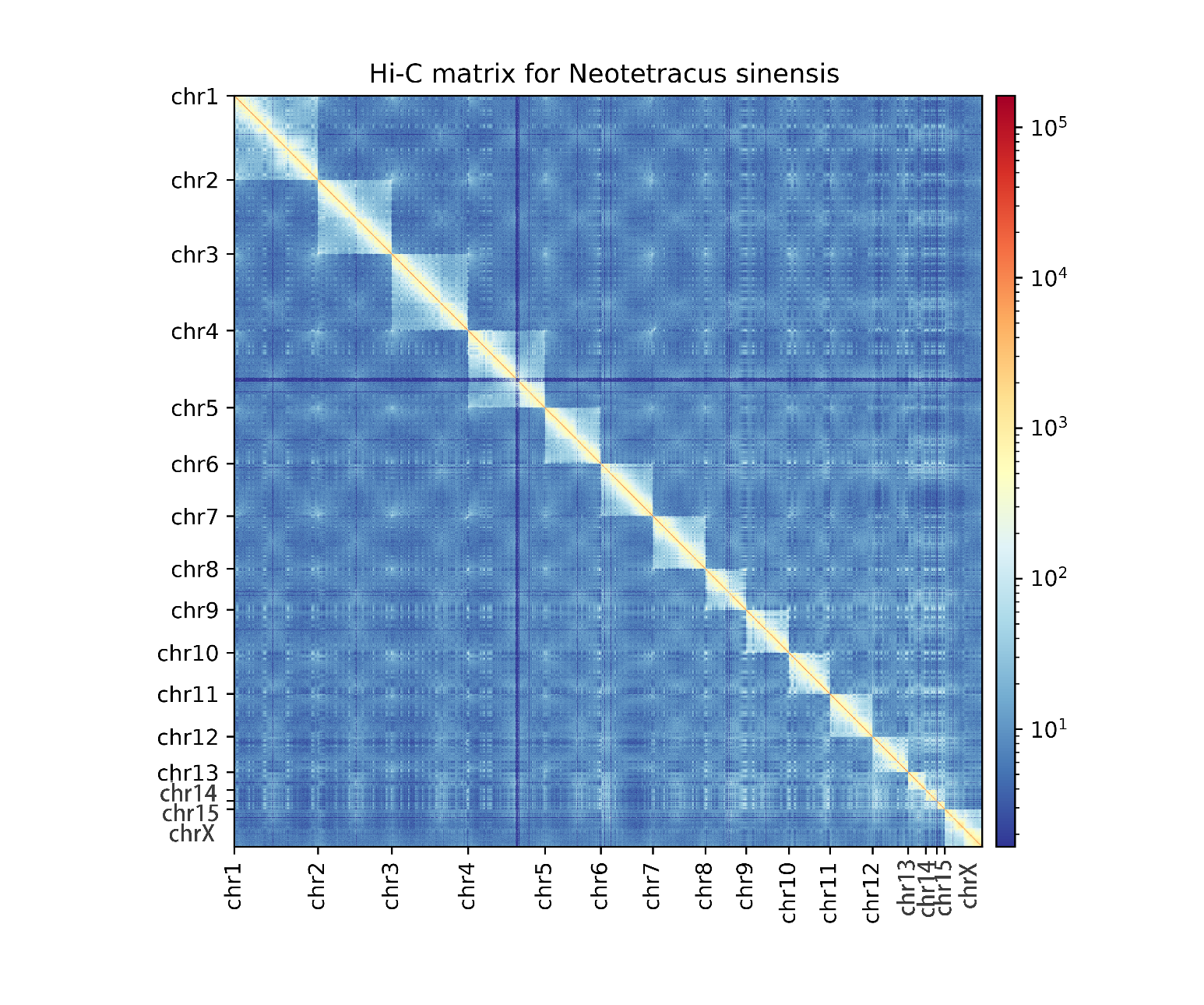
**

**Supplementary Figure S2. Hi-C contact heatmap of the *Neotetracus sinensis* genome assembly.**

The heatmap illustrates the frequency of chromatin interactions across the 16 assembled pseudochromosomes (15 autosomes and the X chromosome) at a 500-kb resolution.


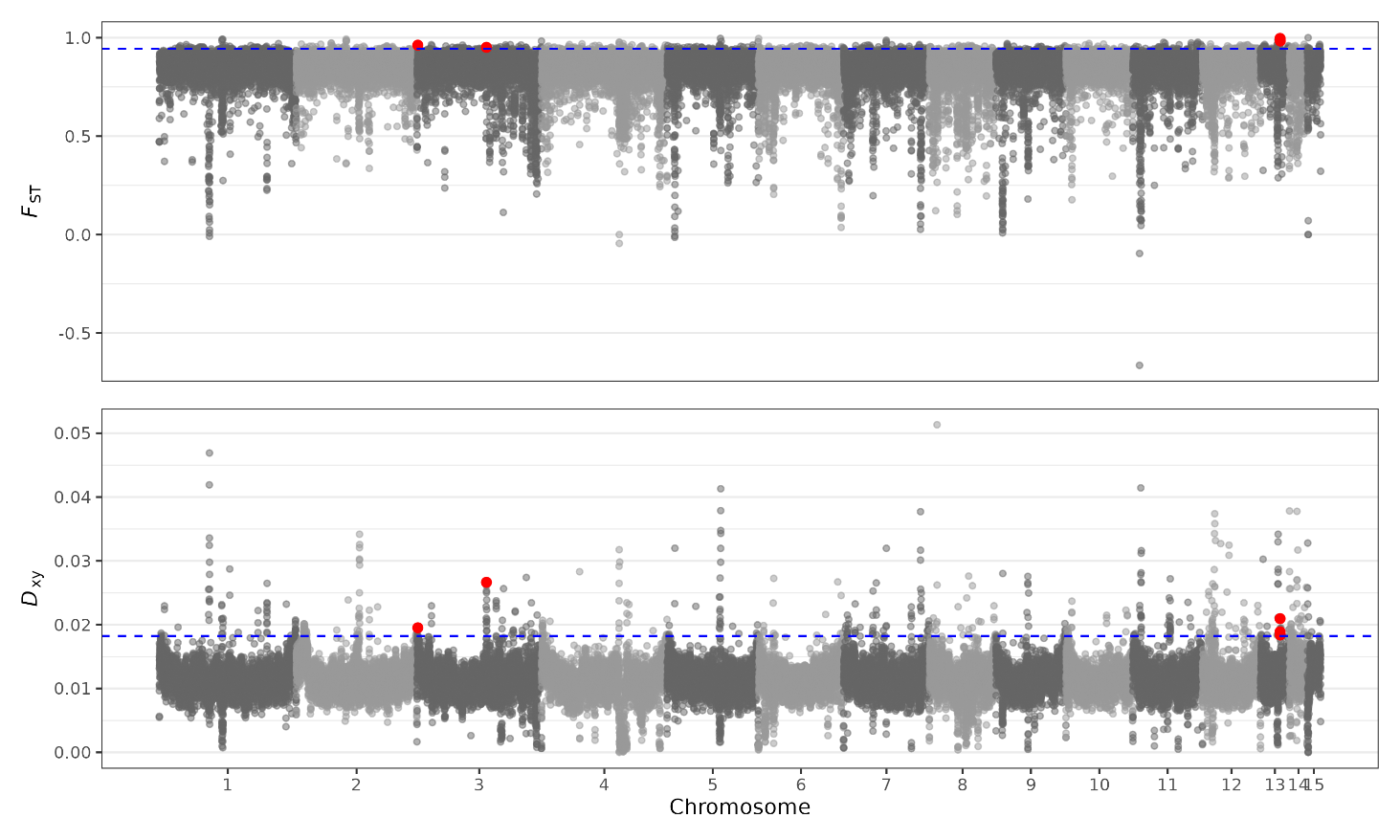
**Fig S3. Genome-wide distribution of genetic divergence metrics (*F*_st_ and *D*_xy_).**

The dual-panel Manhattan plot illustrates the genetic differentiation between the GLG and WL populations across 15 chromosomes. **(A)** The upper panel shows the relative divergence (*F*_st_) calculated in 50-kb sliding windows. **(B)** The lower panel shows the absolute divergence (*D*_xy_) calculated across the same windows. The horizontal blue dashed lines in both panels represent the empirical 99th percentile (top 1%) thresholds. Red dots highlight the "core genomic islands" that reside within the top 1% of both the *F*_st_ and *D*_xy_} distributions simultaneously. These regions represent robust genomic signals of divergence that are less likely to be artifacts of local variations in effective population size.
