# Supplementary material for: Genomic Analysis and Population Divergence Driven by Geographic Isolation in Neotetracus sinensis
Source: Ecol Evol. 2026 Apr 5;16(4):e73375. doi: 10.1002/ece3.73375 (PMC13052205; doi:10.1002/ece3.73375)
Supplement: Supplementary file 2 — Tables S1–S8: ece373375‐sup‐0002‐Tables.docx. [file ECE3-16-e73375-s002.docx]

| SeqNum | SumBase | MinLen | N50Len | MeanLen | MaxLen |
| --- | --- | --- | --- | --- | --- |
| 3,981,878 | 62,231,059,480 | 80 | 15,658 | 15,628.6 | 48,247 |

**TableS1 Statistical summary for PacBio HiFi sequencing dataset**

SeqNum: number of reads;

SumBase: total number of bases;

MinLen, MeanLen, MaxLen,: minimum, average, and maximum read lengths in base pairs;

N50Len: read length N50, defined as the length of the shortest read in the group of longest reads that together make up at least 50% of the total bases.

**TableS2 assembly quality statistics of *Neotetracus sinensis***

| **Quality** | **Metric** | | **Information** |
| --- | --- | --- | --- |
| Continuity | Reads alignment | Mapping rate (%) | 100 |
|  |  | Mean depth (X) | 24 |
|  |  | Coverage rate (%) | 99.99 |
| Base accuracy | Merqury  evaluation | Base pair QV | 61.3 |
|  |  | Error rate (%) | 7.1e-7 |
|  |  | K-mer completeness | 98.2% |
| Functional completeness | Gene | Complete BUSCOs (%) | 95.4 |
|  |  | Complete and single-copy BUSCO (%) | 93.8 |
|  |  | Complete and duplicated BUSCOs (%) | 1.6 |
|  |  | Fragmented BUSC (%) | 0.9 |
|  |  | Missing BUSCOs (%) | 3.7 |
|  |  | Total BUSCO groups search | 9226 |

**TableS3 Summary of repetitive elements identified in the genome assembly of *Neotetracus sinensis***

| **Category** | **Copy number** | **Length(Mb)** | **% of genome** |
| --- | --- | --- | --- |
| Retroelements | 4622986 | 1122.87 | 43.76 |
| SINEs | 3631005 | 747.09 | 29.12 |
| LINEs | 873873 | 342.47 | 13.35 |
| LTR | 118108 | 33.31 | 1.30 |
| DNA transposons | 82069 | 12.60 | 0.49 |
| Unclassified | 684855 | 134.18 | 5.23 |
| Small RNA | 3488364 | 734.13 | 28.61 |
| Simple repeats | 1025436 | 50.15 | 1.95 |
| Low complexity | 124162 | 8.97 | 0.35 |

**TableS4 Proteome assessments summarized from BUSCO and OMArk**

**(A) BUSCO genome completeness annotation** **(mammalia_odb10; proteins mode)**

| **Metric** | **Count** | **Percentage%** |
| --- | --- | --- |
| Complete BUSCOs (C) | 8551 | 92.7 |
| Single-copy (S) | 8441 | 91.5 |
| Duplicated (D) | 110 | 1.2 |
| Fragmented BUSCOs (F) | 305 | 3.3 |
| Missing BUSCOs (M) | 370 | 4.0 |
| Total BUSCO groups searched | 9226 | 100 |

**(B) OMArk completeness on conserved HOGs (reference clade: Laurasiatheria)**

| **Metric** | **Count** | **Percentage%** |
| --- | --- | --- |
| Conserved HOGs (n) | 14,015 | 100 |
| Single | 12,161 | 86.77 |
| Duplicated(total) | 1,416 | 10.10 |
| Unexpected | 1,406 | 10.03 |
| Expected | 10 | 0.07 |
| Missing | 438 | 3.13 |

**(C) OMArk consistency & species composition**

| **Metric** | **Count** | **Percentage%** |
| --- | --- | --- |
| Proteins in proteome | 25,548 | 100 |
| Consistent | 23,233 | 90.94 |
| Partial hits | 1,490 | 5.83 |
| Fragmented | 2,351 | 9.20 |
| Inconsistent | 742 | 2.90 |
| Partial hits | 117 | 0.46 |
| Fragmented | 182 | 0.71 |
| Contaminants | 0 | 0.00 |
| Unknown | 1,573 | 6.16 |

**Main species (OMArk species composition):** *Erinaceus europaeus* (**93.84%**)

**TableS5 Summary of non-coding RNA annotations in the *Neotetracus sinensis* genome**

| **Type** | **Count** | **Total Length** | **Min** | **Max** | **Avg** |
| --- | --- | --- | --- | --- | --- |
| tRNA | 1039 | 88935 | 52 | 418 | 85.6 |
| miRNA | 309 | 24454 | 52 | 112 | 79.1 |
| lncRNA | 109 | 17684 | 38 | 466 | 162.2 |
| rRNA | 345 | 47734 | 63 | 1867 | 138.4 |
| sRNA | 7 | 289 | 32 | 77 | 41.3 |
| snRNA | 3191 | 362753 | 42 | 399 | 113.7 |

**TableS6 Functional annotation coverage of predicted protein-coding genes across databases.**

| **Type** | **Number** | **Percent%** |
| --- | --- | --- |
| eggNOG | 24458 | 95.73 |
| GO | 20095 | 78.66 |
| KEGG | 18578 | 72.72 |
| InterPro | 23102 | 90.43 |
| PANTHER | 22497 | 88.06 |
| Pfam | 21183 | 82.91 |
| UniProt | 23379 | 91.51 |
| Nr | 7648 | 29.94 |

**TableS7 Summary statistics of resequenced *Neotetracus sinensis* genomes**

| **Sample ID** | **Raw data (Gb)** | **Clean data (Gb)** | **Depth (×)** | \| **Mapping rate (%)** \| \| --- \| | **Coverage (%)** |
| --- | --- | --- | --- | --- | --- | --- |
| G48 | 30.91 | 30.49 | 10.02 | 98.38 | 97.59 |
| G49 | 33.06 | 32.62 | 10.77 | 98.22 | 97.68 |
| G50 | 23.24 | 22.94 | 7.52 | 98.50 | 97.13 |
| G52 | 30.90 | 30.47 | 9.78 | 97.44 | 97.52 |
| G53 | 22.20 | 21.88 | 7.00 | 98.02 | 97.14 |
| G54 | 29.24 | 28.85 | 9.56 | 97.69 | 97.57 |
| G55 | 28.78 | 28.45 | 9.37 | 98.37 | 97.48 |
| Wls16 | 35.29 | 34.82 | 11.22 | 97.36 | 99.62 |
| Wls17 | 31.09 | 30.70 | 10.30 | 98.71 | 99.58 |

**TableS8 Summary of SNP counts and heterozygosity in *Neotetracus sinensis* individuals**

| **Sample** | **SNPs** | **Homo** | **Hetero** | **Heterozygosity** | |
| --- | --- | --- | --- | --- | --- |
| G48 | 16,461,386 | 15,146,497 | 1,314,889 | 5.6922E-04 |  |
| G49 | 17,119,005 | 15,755,357 | 1,363,648 | 5.9032E-04 |  |
| G50 | 13,926,925 | 12,995,437 | 931,488 | 4.0324E-04 |  |
| G52 | 15,737,408 | 14,517,535 | 1,219,873 | 5.2808E-04 |  |
| G53 | 15,641,070 | 14,771,230 | 869,840 | 3.7655E-04 |  |
| G54 | 15,661,225 | 14,566,077 | 1,095,148 | 4.7409E-04 |  |
| G55 | 17,313,839 | 16,057,439 | 1,256,400 | 5.4390E-04 |  |
| Wls16 | 4,672,865 | 1,845,137 | 2,827,728 | 1.22412E-03 |  |
| Wls17 | 4,580,012 | 1,551,187 | 3,028,825 | 1.31118E-03 |  |
